# Design, Synthesis, and Characterization of a Novel Tetra-Block Copolymer for High-Performance Self-Healing Batteries

**DOI:** 10.3390/polym17172414

**Published:** 2025-09-05

**Authors:** Işık İpek Avcı Yayla, Omer Suat Taskin, Neslihan Yuca

**Affiliations:** 1 Enwair Energy Technologies Corporation, 34475 Sarıyer, Istanbul, Turkey; iavciyayla@enwair.com; 2 Department of Chemical Oceanography, Institute of Marine Science and Management, Istanbul University, 34134 Fatih, Istanbul, Turkey; 3 Energy Institute, Istanbul Technical University, İTÜ Ayazağa Kampüsü, Enerji Enstitüsü, 34469 Sarıyer, Istanbul, Turkey

**Keywords:** self-healing polymer, silicon anode, tetra-block copolymer

## Abstract

Lithium-ion batteries (LIBs) have become the dominant energy storage technology due to their versatility and superior performance across diverse applications. Silicon (Si) stands out as a particularly promising high-capacity anode material for next-generation LIBs, offering a theoretical capacity nearly ten times greater than conventional graphite anodes. However, its practical implementation faces a critical challenge: the material undergoes a ~300% volume expansion during lithiation/delithiation, which causes severe mechanical stress, electrode pulverization, and rapid capacity decay. In addressing these limitations, advanced polymer binders serve as essential components for preserving the structural integrity of Si-based anodes. Notably, self-healing polymeric binders have emerged as a groundbreaking solution, capable of autonomously repairing cycle-induced damage and significantly enhancing electrode durability. The evaluation of self-healing performance is generally based on mechanical characterization methods while morphological observations by scanning electron microscopy provide direct evidence of crack closure; for electrochemically active materials, electrochemical techniques including GCD, EIS, and CV are employed to monitor recovery of functionality. In this study, a novel self-healing copolymer (PHX-23) was synthesized for Si anodes using a combination of octadecyl acrylate (ODA), methacrylic acid (MA), 2-hydroxyethyl methacrylate (HEMA), and polyethylene glycol methyl ether methacrylate (PEGMA). The copolymer was thoroughly characterized using NMR, FTIR, TGA, SEM, and EDX to confirm its chemical structure, thermal stability, and morphology. Electrochemical evaluation revealed that the PHX-23 binder markedly improves cycling stability, sustaining a reversible capacity of 427 mAh g^−1^ after 1000 cycles at 1C. During long-term cycling, the Coulombic efficiency of the PHX-23 polymer is 99.7%, and similar functional binders in the literature have shown similar results at lower C-rates. Comparative analysis with conventional binders (e.g., PVDF and CMC/SBR) demonstrated PHX-23’s exceptional performance, exhibiting higher capacity retention and improved rate capability. These results position PHX-23 as a transformative binder for silicon anodes in next-generation lithium-ion batteries.

## 1. Introduction

Functional polymers represent a class of advanced materials engineered to exhibit tailored chemical, physical, or biological properties, enabling their utilization in specialized applications. These macromolecular systems demonstrate unique characteristics—such as stimuli responsiveness [1,2] to pH, temperature, or light; electrical conductivity; or self-healing capabilities [3,4]—rendering them indispensable in interdisciplinary research. Key applications include biomedical fields, advanced electronics, and sustainable technologies. Their programmable functionality and adaptability continue to drive innovations in materials science and engineering. In addition, composites are prepared to improve the functional polymer’s uses and properties. Functional polymer composites improve material properties by adding fillers such as carbon fibers [5,6], carbon nanotubes [3,7,8], graphene [9,10], or nanoparticles. These additives improve strength, thermal stability, and electrical conductivity, making polymers suitable for aerospace, electronics, and automotive applications.

Self-healing polymers are smart materials capable of autonomously repairing mechanical damage through intrinsic (reversible bonds like Diels-Alder) or extrinsic (e.g., embedded microcapsules with healing agents) mechanisms. Their synthesis often involves incorporating dynamic covalent bonds (disulfides or hydrogen bonds) or phase-separated architectures to enable recovery. These polymers are synthesized via methods [11,12,13] such as free-radical polymerization, controlled polymerization (RAFT or ATRP), or sol–gel processes, with applications ranging from aerospace coatings to biomedical implants.

Free-radical polymerization plays a crucial role in the development of self-healing polymers due to its ability to form highly functional networks that can undergo reversible chemical reactions. In self-healing materials, free radicals initiate polymerization at specific sites, enabling the material to repair itself through hydrogen bonding and hydrophobic interaction when damage occurs [13,14]. The reactive sites can bond with monomers or other functional groups, restoring the polymer’s structural integrity. This process allows the material to recover its original properties without the need for external intervention, making free radical polymerization an essential method for designing efficient self-healing systems.

Silicon (Si) is regarded as a highly promising next-generation anode material for lithium-ion batteries (LIBs), centered to replace conventional graphite and Li_4_Ti_5_O_12_, owing to its elevated theoretical gravimetric capacity (3500 mAh g^−1^), the storage capacity of a single silicon atom which accommodates 4.4 lithium atoms and its abundance in the Earth’s crust, low cost, and non-toxicitiy [15,16]. However, because of significant volume changes (>300% expansion) that result in an unsteady solid–electrolyte interface (SEI) and induced pulverization during charge/discharge, the current Si anode continues to experience rapid capacity decline and battery failure [17,18,19]. To address these issues, several kinds of Si nanostructures have been tested in the last ten years, such as hollow nanospheres [20], yolk–shell structures [21], nanowires [22], nanotubes [23] and nano-/micro-structured silicon–graphite composites [24]. To address these challenges, some studies are also being conducted on a variety of polymer binders in addition to these different nanostructures. In polymer binder studies, strong mechanical strength and adhesion strength binders such as polyacrylic acid [25], sodium carboxymethyl cellulose [26,27], alginate [27,28] and gum arabic [29], cross-linked binders [30,31,32,33,34], functional binders [35,36,37,38,39,40] and self-healing binders [41,42,43,44,45] have been used.

Self-healing polymers play a pivotal role in mitigating the mechanical degradation of Si anodes, thereby enhancing both electrochemical performance and cycle life in lithium-ion batteries (LIBs) [42,43,46]. During cycles of charging and discharging, the volume of silicon anodes has a tendency to expand and contract, which can result in cracking and capacity loss.

By incorporating self-healing polymers, the composite material can autonomously repair microcracks, preserving the structural integrity of the silicon anode and consequently improving the battery’s cycling efficiency and long-term stability. This self-repair mechanism helps mitigate degradation, ensuring better performance and a longer lifespan for lithium-ion batteries.

This study presents design, synthesis, systematic optimization, and comprehensive characterization of self-healing tetra-block copolymer (SHP). Structural optimization of the polymers was conducted by modulating critical reaction parameters, including polymerization duration, monomer concentrations, and monomer utilization efficiency. The chemical properties of the SHP systems were rigorously characterized via NMR, FTIR, TGA/DSC, SEM, and EDX. Furthermore, the electro-chemical performance of silicon-based anodes was evaluated through galvanostatic charge–discharge cycling using CR2032-type half-cells with lithium metal counter electrodes.

## 2. Materials and Methods

Details regarding the materials used and characterization methods are provided in the Supplementary Materials.

### 2.1. Synthesis of Tetra-Block Copolymer

In the polymerization, C18, HEMA, PEGME, and MA are dissolved in 25 mL chlorobenzene at room temperature. DBO is added as initiator to the homogeneous monomer solution and the solution is heated and stirred for specified time under nitrogen atmosphere. Molarity of monomers, reaction temperature, and time for copolymerization are summarized in Table S1. Purification was performed using the precipitation method with hexane at the end of the reaction. After precipitation, the powdered polymer was vacuum-dried for 24 h at 60 °C. (Mn, NMR: 2479.75 g/mol).

### 2.2. Electrode Preparation and Battery Assembling

The content of the slurry is 60 wt.% Si nanoparticles and 10 wt.% carbon black, 10 wt.% CNT and 20 wt.% polymer binder. First, polymer was homogenized in N-methyl-2-pyrrolidone (NMP) and CNT, CB, and Si were added. The mixture was blended using a magnetic stirrer for 5 h, then an ultrasonic homogenizer for 1 h to prevent the agglomeration of Si nanoparticles, and the slurry was covered to copper foil by a doctor blade coater. These electrodes were dried at 60 °C for 1 h, 110 °C for 4 h, and then at 140 °C for another 1 h under vacuum. The loading mass of Si is measured as 0.3–0.7 mg/cm^2^.

The electrodes were used to assemble the coin cells (CR2032) while Li metal was used as a counter electrode. Polypropylene separators and the electrolyte consisting of 1 M LiPF_6_ in EC:DEC (1:1 *w*/*w*) were added. All the coin cells were assembled in the Ar-filled glovebox.

## 3. Results

**Scheme 1:**
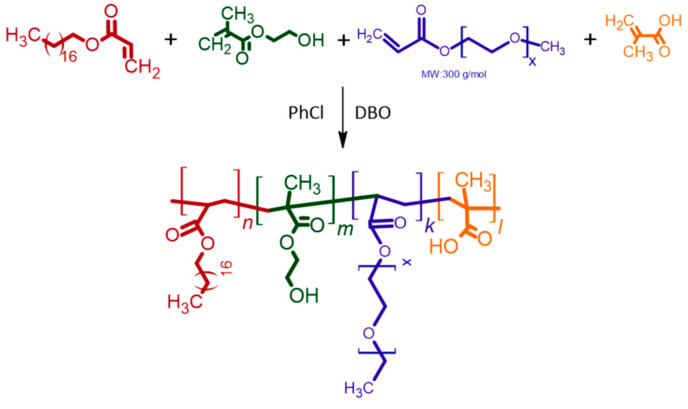
Synthesis of PHX via free-radical polymerization.

Monomers with functional groups were polymerized using the free radical polymerization method. The reaction scheme is given Scheme 1.

**Figure 1:**
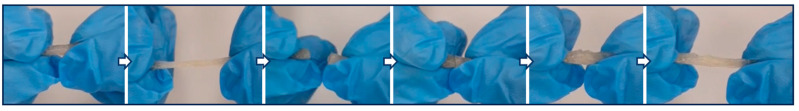
Self-healing test for PHX-23 polymer.

In the experiments presented in Table S1, the PHX-1, 2, 3, and 4 polymers persisted in gel form due to intramolecular esterification and could not be purified. The effect of reaction time was investigated in the syntheses numbered PHX-5-9. It was observed that gelation began at the end of the sixth hour of the reaction. The purified powder products from PHX-5-9 were found to be dispersible in NMP but soluble in EC:DEC. To obtain a denser structure, the reaction temperature was elevated to 90 °C, resulting in the powders from experiments PHX-10-11 being insoluble and undispersible in any solvent. Due to their indispersibility in NMP, they were unsuitable for use as binders. In the syntheses of PHX-12, 13, 14, 16, 17, and 24, purification did not provide any powder product. The PHX-15 and 19 polymers persisted in gel form due to intramolecular esterification and could not be purified. PHX-20, 21, 22, and 23 effectively synthesized and purified as self-healing polymer. The PHX-20, 21, 22, and 23 polymers were used to prepare the electrodes, and their interactions with the electrolyte were investigated. In the electrodes prepared in PHX-20 and 21, deformation of the coating was observed after wetting with electrolyte. When electrodes prepared by the PHX-23 and PHX-24 self-healing binders, it was found that the electrode with a PHX-23 binder became wet, whereas the electrode with PHX-24 remained dry. The improved wetting of PHX-23 can be attributed to the tetra-block copolymer, which, synthesized through careful adjustment of monomer ratios and reaction time, adopts a more regular and linear structure, thereby facilitating enhanced chain packing, promoting self-assembly, and contributing to the overall structural regularity of the material. Thus, the PHX-23 polymer was chosen for coin cell application and polymer characterization. Self-healing test images for PHX-23 are shown in Figure 1. A video of this self-healing test is also provided in the Supplementary Materials. The self-healing capability of the copolymer can be ascribed to the synergistic contribution of two non-covalent interactions: hydrophobic association among the long hydrocarbon chains of the octadecyl acrylate segments, and hydrogen bonding interactions arising from the –OH groups distributed along the polymer backbone. Together, these reversible interactions enable the polymer chains to reconnect across fractured interfaces, thereby imparting efficient and repeatable healing behavior.

**Figure 2:**
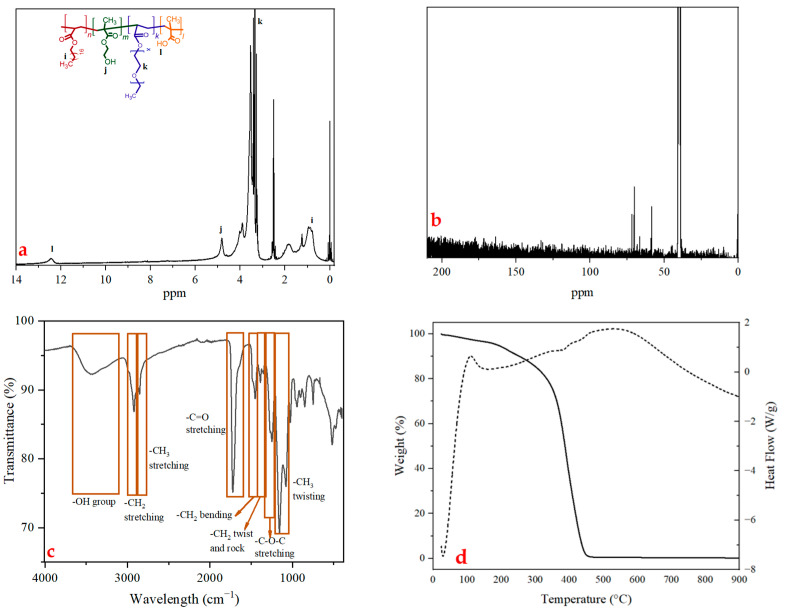
(**a**) ^1^H, (**b**) ^13^C NMR, (**c**) FTIR, and (**d**) TGA-DSC results of PHX-23.

The tetra-block copolymer structure was verified by ^1^H and ^13^C NMR spectroscopy, with clear detection of characteristic proton and carbon resonances for both constituent units (Figure 2). In the ^1^H NMR spectrum (Figure 2a), the OH group of methacrylic acid in the tetra-block copolymer was responsible for the low-intensity peak at 12.4 ppm [47]. The OH of HEMA was responsible for the peak at 4.8 ppm [48]. One might state that the broad, powerful peak at 3.5 ppm was a part of the PEG acrylate repeating unit [49]. Lastly, it was observed that C18 is responsible for the methylene group peak at 0.9 ppm [50]. In Figure 2b, the signal of the C-C bonds of C18 at 39.1 to 40.8 ppm and the peak corresponding to the C-O-C and C-OH bonds appearing at 70.3 to 71.9 ppm and 58.6 ppm, respectively, evidenced the expected structure [48,49,51,52]. The molecular weight of the PHX-23 polymer was calculated to be 2479,75 g/mol by ^1^H-NMR.

The chemical structure of PHX-23 was approved by FTIR, as shown in Figure 2c. The spectrum exhibited characteristic absorption bands corresponding to key functional groups. A broad peak in the range of 3600–3200 cm^−1^ was attributed to O-H stretching vibrations, indicating the existence of hydroxyl (–OH) groups, which coming from HEMA and MA monomers. The characteristic aliphatic C-H stretching vibrations were observed at 2920 cm^−1^ (asymmetric-CH_2_ stretch) and 2850 cm^−1^ (symmetric-CH_3_ stretch), confirming the incorporation of hydrocarbon chains in the polymer backbone. A strong, sharp peak at 1720 cm^−1^ corresponded to C=O stretching, suggesting the existence of carbonyl groups, likely from ester or carboxylate functionalities from all monomers. The bending vibrations of the –CH_2_ groups appeared at 1452 cm^−1^, while the –CH_2_ twisting and rocking modes were detected at 1384 cm^−1^, further supporting the aliphatic nature of the polymer. The region between 1155 and 1080 cm^−1^ displayed prominent peaks belonging to C–O–C stretching vibrations, typical of ether or ester linkages, along with –CH_3_ twisting motions, reinforcing the proposed polymer structure. These FTIR findings were further corroborated by NMR spectroscopy (^1^H and ^13^C), which provided complementary evidence for the polymer’s molecular architecture as reported in previous studies [53,54,55,56]. The consistency between FTIR and NMR data strongly validates the successful synthesis of the target polymer.

PHX-23’s thermal stability was investigated using TGA. The TG and DSC curves for PHX-23 under nitrogen are given in Figure 2d. The PHX-23 polymer started degrading at 250 °C, with 392 °C identified as the degradation temperature. According to the DSC result, the glass transition temperature (T_g_) was found to be 112 °C. The DSC profile of PHX-23 indicated that the material is entirely degraded at 524 °C. The material exhibited relatively low thermal stability in TGA analysis, with degradation occurring at lower temperatures, consistent with its low molecular weight as determined by NMR spectroscopy. The degradation onset of ~250 °C does not pose a limitation for electrode processing, as the drying step for solvent removal is typically conducted at 80–120 °C, which is significantly lower than the degradation threshold of PHX-23. Under these conditions, the polymer retains its structural integrity and functional groups without undergoing thermal decomposition, thereby ensuring that its interfacial adhesion, self-healing capability, and overall binder performance are preserved throughout electrode fabrication. Therefore, despite its relatively lower thermal stability compared to some conventional binders, PHX-23 remains fully adequate for practical processing and operation. Only 0.5% char residue remained after non-oxidative degradation in an inert medium, consistent with the material’s aliphatic structure. Polymers and copolymers derived from analogous monomer systems have demonstrated comparable thermal behavior in prior studies [57,58]. To enhance thermal resistance while preserving structural similarity, increasing the molecular weight is essential. This can be achieved by optimizing synthetic parameters, including reaction temperature, polymerization duration, and cross-linking density.

**Figure 3:**
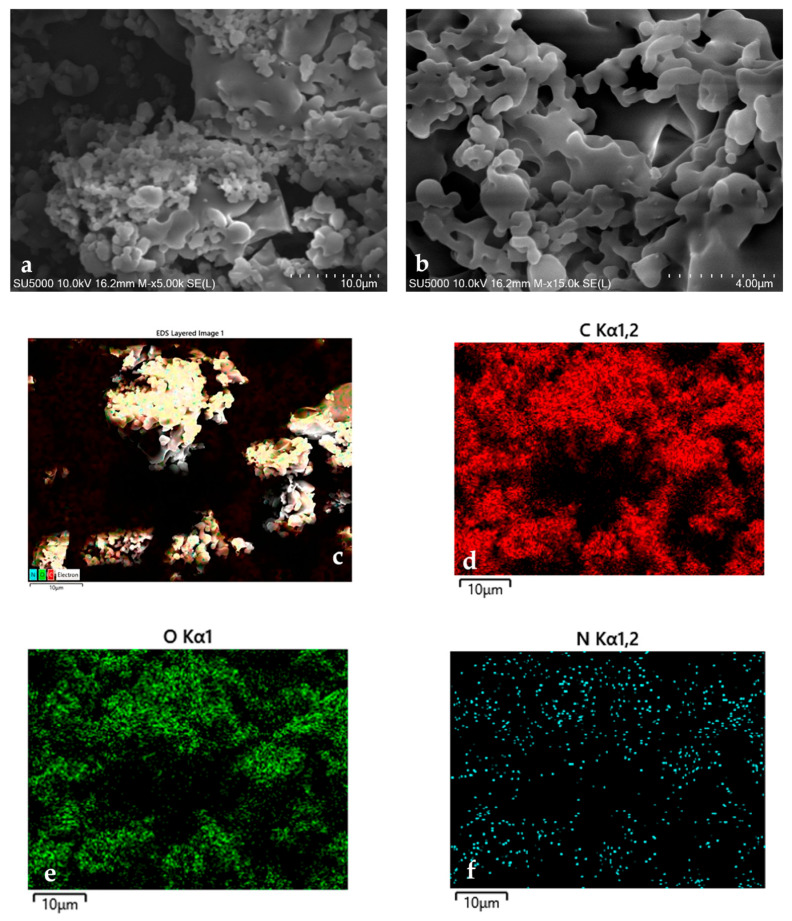
(**a**) 5.0k and (**b**) 15.0k SEM images, (**c**) all elements, (**d**) C, (**e**) O, and (**f**) N elements EDX of PHX-23.

PHX-23’s surface morphology and microstructure were characterized by SEM, as depicted in Figure 3a,b in two different scale bars (4 and 10 μm). The SEM images revealed a highly porous architecture with interconnected voids. This porous morphology of PHX-23 provides effective mechanical buffering to accommodate large volume fluctuations of Si during cycling, while promoting more uniform SEI formation that enhances interfacial stability and prolongs electrochemical performance. Such multifunctional advantages of porous binder systems have been consistently reported in silicon anodes, showing improved stress tolerance and cycling durability [59,60].

EDX was utilized to analyze the elemental composition and spatial distribution within the polymer matrix (Figure 3c–f). The EDX mapping confirmed the uniform distribution of carbon (C), oxygen (O), and nitrogen (N) atoms throughout the polymer matrix, indicating a homogeneous chemical structure with no significant phase segregation.

**Figure 4:**
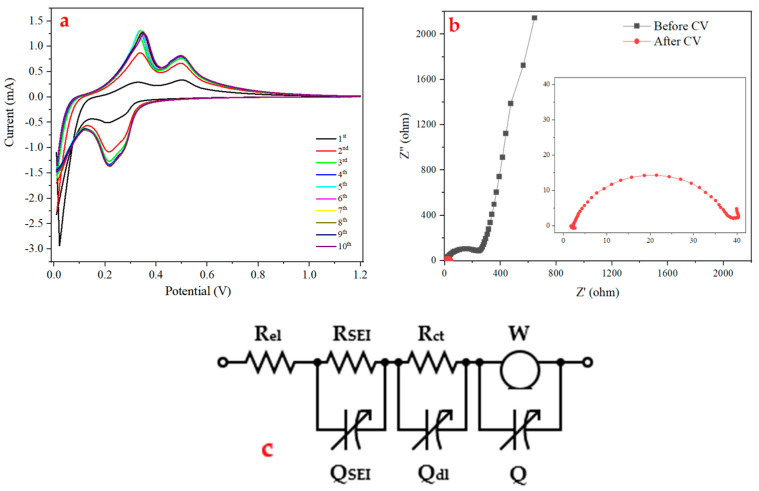
(**a**) CV curves obtained at a scan rate of 0.05 mV/s within a voltage range of 0.01 to 1.2 V, (**b**) Nyquist plots (before and after CV), and (**c**) equivalent circuit form Nyquist plot of Si/PHX-23 electrode.

The lithium insertion/extraction mechanisms were characterized by cyclic voltammetry (CV) (Figure 4a) using a scan rate of 0.05 mV s^−1^ over the 0–1.2 V potential window. The difference between the first cycle shown in the figure and the subsequent cycles is thought to be due to a “formation” effect associated with the coating of the electrode during the first Li discharge [61]. After the first cycle, the cycles had taken on a generally repeatable pattern. Similarly to the literature, the two anodic peaks at 0.33 V and 0.49 V seen in the CV are attributed to the partial decomposition of the highest lithiated phase Li_4.2_Si_12_ and the complete removal of Li from silicon [62,63]. According to CV measurements, the phase transition between Li_3.16_Si and Li_7_Si_3_ was represented by the first anodic peak seen, whereas the phase transition between Li_7_Si_3_ and LiSi was associated with the second anodic peak. The lithiation process with amorphous Si generated during phase transitions was associated with a new cathodic peak that was detected at about 0.2 V [64]. The stabilized cathodic current corresponding to lithium insertion and the anodic current associated with deinsertion indicate that the formed SEI layer stabilizes starting from the fourth cycle. This behavior can be attributed to the chemical architecture of PHX-23, which consists of four distinct blocks providing abundant functional groups capable of interacting with the Si surface. These functionalities promote uniform SEI formation by anchoring electrolyte decomposition products and minimizing continuous side reactions. In contrast, conventional binders such as CMC/SBR or PVDF primarily serve as mechanical matrices with limited interfacial chemistry; PVDF, for instance, is largely inert, while CMC/SBR offer fewer active binding sites. The synergistic contribution of hydrogen bonding, hydrophobic interactions, and inter-chain adhesion in PHX-23 helps to maintain intimate contact between the binder and Si particles, thus suppressing unstable SEI growth and leading to a more stable electrochemical interface.

EIS measurements were performed both before and after the CV with the aim to better understand how the polymer affected Si anodes. The EIS result of the PHX–silicon electrode at before and after the CV test is given in Figure 4b. The Nyquist plot displays a single, moderately sized semicircle in the high-frequency region, followed by a 45° Warburg slope at mid-to-low frequencies. While charge transfer resistance was seen in the mid-frequency range, intrinsic electrode resistance was found in the low-frequency range. Lithium diffusion was linked to a 45° line slope in the low-frequency range and this line is significantly reduced after CV because of SEI [65]. Also, the charge transfer resistances (R_ct_) were decreased after CV. Since the increased volume of silicon during lithiation improved the electrical connection between the active material and conductive carbon particles, the decrease in R_ct_ that is seen after conversion is an expected result [66]. In addition, the incorporation of a self-healing polymer binder significantly reduced R_ct_ by 86% after cycling, attributable to its dual functionality: (i) autonomous repair of electrode microcracks and (ii) effective maintenance of conductive pathways between active material (Si) and carbon additives throughout cycling.

An equivalent circuit diagram of the Si/PHX-23 electrode is given in Figure 4c. In this diagram, the ionic resistance of the cell’s electrolyte is represented by R_el_, which is in the high-frequency region. Also, R_sei_ and Q_sei_ are in the high-frequency region and they represent the resistance and capacitance of ions passing through the surface layer, respectively. R_ct_ and Q_dl_ are, respectively, the charge transfer resistance and double-layer capacitance in the intermediate frequency range. A diffusion process in the solid/liquid phase that is controlling in the low-frequency region was represented by the Warburg (W) impedance. The insertion or desertion capacitance (Q) represented the lithium occupation process in the lattice. There was a connection between Q and W because it is related to the movement of lithium ions [67]. Therefore, it was shown as connected in parallel in the equivalent circuit.

**Figure 5:**
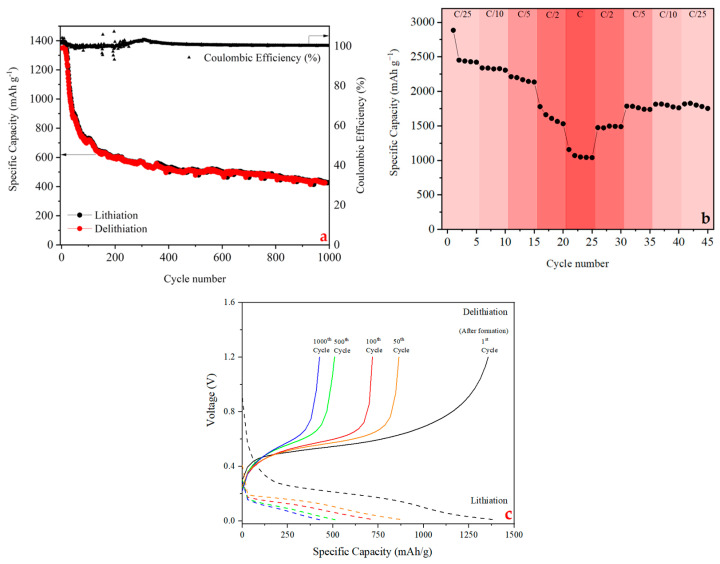
(**a**) GCD and coulombic efficiency at 1C, (**b**) C-rate test, and (**c**) voltage profile at 1C of Si/PHX-23 electrode.

A galvanostatic charge–discharge (GCD) test was performed and is given in Figure 5a. It was found that, due to the high resistance of the SEI created by polymer, the electrodes could not reach the theoretical capacity of the Si nanoparticles (3500 mAh g^−1^). The PHX-23 polymer electrodes showed an initial capacity of 2885 during the first formation cycle, and after the formation cycle, the electrode showed 1390 mAh g^−1^ at 1C according to active material weight. During galvanostatic cycling, the silicon anode experienced significant capacity degradation caused by structural pulverization and excessive dendrite growth. The incorporation of the PHX-23 polymer binder effectively addressed these issues due to its intrinsic self-healing capability, which preserved electrode integrity and outperformed conventional binders. Over repeated cycles, the PHX-23-based electrode demonstrated remarkable electrochemical stability, exhibiting minimal capacity loss, consistent charge/discharge plateaus, and reduced polarization. These findings highlight the superior cyclability and reversibility enabled by the PHX-23 binder system. At the end of 1000 cycles at a 1C rate, the capacity of the Si/PHX-23 polymer electrode was found to be 427 mAh g^−1^. The stability of the SEI layers and the slower rate of electrolyte degradation on the Si surface during cycles can be seen by the high coulombic efficiencies of the Si electrode.

To determine the advantage of PHX-23, electrodes were prepared using the same formulation as CMC-SBR, which is commonly used. The charge–discharge characteristics of the prepared CMC-SBR electrode at C/5 rate are displayed in Figure S1. As can be seen, CMC-SBR binder provided a capacity of 502 mAh g^−1^ after 500 cycles at C/5. Based on these results, it was determined that PHX-23 exhibits superior cycle performance compared to CMC-SBR.

Figure 5b presents the rate capability of the silicon anode across multiple C-rates (C/25 to 5C), where the initial activation cycle was performed at C/25. According to electrochemical theory, rate performance is the transfer of a specified quantity of charge while keeping the cell voltage limit constant. Internal cell resistance under current load generates overpotential, which simultaneously reduces both the achievable specific capacity and operational voltage window of the battery. Consequently, even though the current density increased steadily from C/25 to 1C, the Si/PHX-23 electrode displayed limited capacity at high C ratios (>1C). After switching from high C ratios to low C ratios, the Si anode kept operating normally.

In Figure 5c, the voltage curves of the Si/PHX-23 electrode at different cycles are presented. During discharge, the flat voltage plateau below 0.45 V indicates a thermodynamically favorable Li-Si alloying reaction. Si undergoes gradual amorphization as Li^+^ ions insert into its structure, forming non-crystalline Li_x_Si_γ_ alloys. The length of the plateau reflects the extended phase transformation from crystalline Si to amorphous Li-Si alloys. The pulverization of active materials caused the voltage plateau to decrease from cycle to cycle.

**Figure 6:**
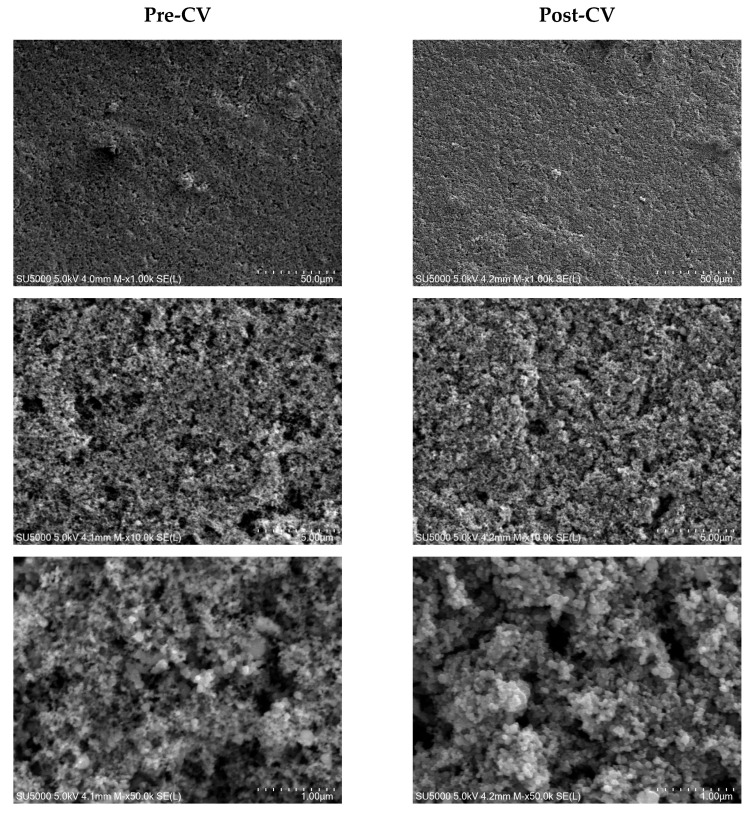
SEM images of pre-CV and post-CV form of PHX-Si electrode.

SEM images of the Si/PHX-23 electrode before and after cycling at magnifications of 1.0k, 10k, and 50k are shown in Figure 6. Figure 6 presents the morphological evolution of the Si/PHX-23 electrode through SEM characterization at three magnifications (1.0k×, 10k×, and 50k×), comparing to a pristine electrode and post-CV electrode. Electrode integrity and electron transport are improved by the uniform dispersion of CNT. Thanks to the SHP property of the PHX-23 polymer, it can reduce the internal stress and make it possible to tolerate the volume change of silicon, as seen in the SEM image, where there are no cracks on the electrode after CV. Pre- and post-cycle comparative SEM analyses revealed a transformation from the crystalline to the amorphous phase in the silicon particles after the cycle.

**Figure 7:**
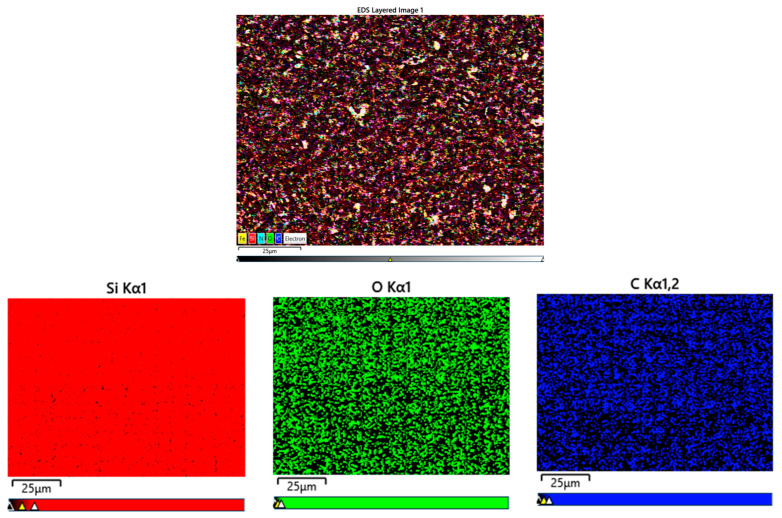
EDX images of Si/PHX-23 electrode.

According to the EDX elemental mapping images presented in Figure 7, it is evident that carbon (C), oxygen (O), and Si are uniformly distributed across the electrode surface. This homogeneous dispersion confirms the effective integration of the PHX-23 polymer binder with the silicon active material, suggesting a well-engineered composite electrode structure.

## 4. Conclusions

With its exceptional theoretical capacity, Si stands as the most promising next-generation anode material for high-energy-density lithium-ion batteries. However, rapid capacity loss results from Si’s significant volume expansion during charge/discharge cycles. In order to mitigate the negative effects of volume changes, Si-based anodes need to be modified structurally or materially. Self-healing polymers are one example of an innovative solution that can assist offset these volume variations and enhance cycling stability. In this study, PHX-23 was generated via a free copolymerization procedure and applied as the binder for the production of the silicon anode. Although PHX-23 has so far been synthesized only on a laboratory scale, its monomers are commercially available and the synthesis involves a straightforward free-radical polymerization, which is in principle compatible with large-scale processing. While it is not yet economically competitive with established binders such as PVDF or CMC, this work should be regarded as a proof-of-concept for a self-healing binder strategy. Future studies focusing on reaction optimization, cost-efficient synthesis pathways, and environmentally benign processes will be essential to assess the scalability and industrial viability of PHX-23 for practical silicon anode applications. FTIR and NMR were used to characterize the chemical structure of PHX-23, and the spectra showed all of the polymer’s unique peaks. Furthermore, thermal behavior of PHX-23 was determined by DSC/TGA. By using SEM and EDX analysis, the porous structure and uniform distribution of elements in PHX-23 were determined. The homemade cut-and-paste technique has also been used to confirm PHX-23’s capability for self-healing. The Si/PHX-23 electrode delivers a high reversible capacity of 427 mAh g^−1^ after 1000 cycles at a 1C. Although the galvanostatic charge–discharge profiles show a decrease from an initially high capacity (2885 mAh g^−1^) to 427 mAh g^−1^ after 1000 cycles at 1C, this value remains above the practical capacity of commercial graphite anodes (~372 mAh g^−1^). This indicates that, despite inevitable fading associated with the large-volume expansion of silicon, the binder design enables the electrode to retain a practically useful capacity beyond that of conventional carbonaceous anodes. Therefore, the results should not be interpreted as an immediate replacement of graphite, but rather as evidence that the self-healing binder strategy can mitigate degradation mechanisms and extend the practical utility of Si anodes toward real-world applications. Post-cycling morphological and electrochemical analyses demonstrate that the PHX-23 binder effectively repairs cracks induced by silicon’s significant volume expansion, thereby preserving electrode structural integrity. This self-healing functionality, combined with the binder’s robust mechanical strength, offers a promising strategy to mitigate degradation in silicon-based anodes. This innovative binder design effectively accommodates volume fluctuations while enabling the development of high-energy-density lithium-ion batteries with extended cycle lives. In addition, while lower molecular weight (Mw) polymers exhibit enhanced processability, future research should explore the synthesis of higher Mw variants for applications where solubility is not a primary requirement, as increased chain length may improve mechanical flexibility while maintaining structural integrity.

## Data Availability

The original contributions presented in this study are included in the article. Further inquiries can be directed to the corresponding authors.

## Supplementary Materials

The following supporting information can be downloaded at: https://www.mdpi.com/article/10.3390/polym17172414/s1, Materials and Methods, Figure S1: GCD at C/5 of Si/CMC-SBR electrode; Table S1: Molarity of monomers, reaction temperature, and time for copolymerization; Video S1: Self-healing test of PHX-23.
