# Supplementary material for: Design, Synthesis, and Characterization of a Novel Tetra-Block Copolymer for High-Performance Self-Healing Batteries
Source: Polymers (Basel). 2025 Sep 5;17(17):2414. doi: 10.3390/polym17172414 (PMC12431371; doi:10.3390/polym17172414)
Supplement: Supplementary file 1 [file polymers-17-02414-s001.zip › Supplementary data_rv1.pdf]

## Supplementary data

# Design, Synthesis, and Characterization of a Novel Tetra-Block Copolymer for High-Performance Self-Healing Batteries

## Materials and Methods

*Materials*

Octadecyl acrylate (C18), 2-hydroxy ethyl methacrylate (HEMA), methacrylic acid (MA), polyethyleneglycol methyl ether methacrylate (PEGME), dibenzoyl peroxide (DBO) and chlorobenzene (PhCl) were received from Sigma-Aldrich. Nanosilicon powder (Si) (particle size is 30–50 nm, Nanostructured and Amorphous Materials, Inc.) was used as the anode active material. Lithium-ion electrolyte was received from Gotion, including 1.0 M $LiPF_6$ in ethylene carbonate and diethyl carbonate (EC:DEC= 1:1 w/w). Carbon nanotube solution (CNT) and carbon black (CB) were purchased from Cone Science and MTI Corp. respectively.

*Synthesis of Tetra-Block Copolymer*

**Table S1.** Molarity of monomers, reaction temperature and time for copolymerization.

| Exp. No | Molarity (M) | | | | | T (°C) | t (h) |
|---|---|---|---|---|---|---|---|
| | C18 | HEMA | MMA | PEGME | DBO | | |
| PHX-1 | 0.62 | 2.4 | 1.2 | 1.12 | 0.018 | 70 | 24 |
| PHX-2 | 0.62 | 2.4 | 1.2 | 1.12 | 0.018 | 70 | 6 |
| PHX-3 | 0.62 | 2.4 | 0.02 | 1.12 | 0.018 | 70 | 1 |
| PHX-4 | 0.62 | 2.4 | 1.2 | 1.12 | 0.018 | 70 | 2 |
| PHX-5 | 0.124 | 0.48 | 0.24 | 0.224 | 0.0036 | 70 | 2 |
| PHX-6 | 0.124 | 0.48 | 0.24 | 0.224 | 0.0036 | 70 | 6 |
| PHX-7 | 0.124 | 0.48 | 0.24 | 0.224 | 0.0036 | 70 | 24 |
| PHX-8 | 0.124 | 0.48 | 0.24 | 0.224 | 0.0036 | 70 | 72 |
| PHX-9 | 0.124 | 0.48 | 0.24 | 0.224 | 0.0036 | 70 | 7 |
| PHX-10 | 0.124 | 0.48 | 0.24 | 0.224 | 0.0036 | 90 | 96 |
| PHX-11 | 0.124 | 0.48 | 0.24 | 0.224 | 0.0036 | 90 | 24 |
| PHX-12 | 0.124 | 0.12 | 0.12 | 0.224 | 0.0036 | 70 | 24 |
| PHX-13 | 0.248 | 0.12 | 0.12 | 0.224 | 0.0036 | 70 | 24 |
| PHX-14 | 0.496 | 0.12 | 0.12 | 0.224 | 0.0036 | 70 | 24 |
| PHX-15 | 0.62 | 0.60 | 0.60 | 1.12 | 0.018 | 70 | 24 |
| PHX-16 | 1.24 | 0.60 | 0.60 | 1.12 | 0.018 | 70 | 24 |
| PHX-17 | 0.248 | 0.48 | 0.12 | 0.224 | 0.0036 | 80 | 24 |
| PHX-18 | 0.496 | 0.48 | 0.12 | 0.224 | 0.0036 | 80 | 24 |
| PHX-19 | 0.062 | 0.48 | 0.24 | 0.224 | 0.0036 | 80 | 24 |
| PHX-20 | 0.031 | 0.48 | 0.24 | 0.896 | 0.0036 | 80 | 24 |
| PHX-21 | 0.031 | 0.48 | 0.24 | 0.896 | 0.0036 | 80 | 72 |
| PHX-22 | 0.031 | 0.48 | 0.24 | 0.896 | 0.0036 | 80 | 18 |
| PHX-23 | 0.0155 | 0.24 | 0.12 | 0.448 | 0.0018 | 80 | 48 |
| PHX-24 | 0.00775 | 0.12 | 0.06 | 0.224 | 0.0009 | 80 | 48 |

*Characterization*

$^{1}H$ and $^{13}C$ nuclear magnetic resonance (NMR) spectra were taken in $(CD_3)_2SO$ with $Si(CH_3)_4$ as standard at 300 MHz at room temperature (Bruker Biospin). The molecular weight of the polymer was calculated from H-NMR spectrum. FTIR measurements were performed by PerkinElmer Spectrum Two FTIR system. TGA measurements were performed in nitrogen atmosphere with a flow rate of 10°C/min at 0-1200°C by TA Instruments SDT 650 Simultane DSC/TGA. SEM and EDX images of the polymer and electrode surface were collected with a Hitachi SU-5000 scanning electron microscope with an accelerating voltage of 5 kV using the high vacuum mode at room temperature.

The performance of the assembled 2032-coin cells was evaluated with Neware Battery Test system at room temperature. The cut-off voltage of cell testing is between 1.2 V and 0.01 V, assuming a theoretical value of 3500 mAh/g for Si. Galvanostatic tests were performed at C/25, C/5, C/10 and C/2 for 3 cycles and 1C for 1000 cycles. The C-rate tests were performed at C/25, C/10, C/5, C/2, 1C for 5 cycles each at the same voltage range. Cyclic voltammetry (CV) and electrochemical impedance spectroscopy (EIS) analysis are performed at GAMRY Interface 1010E potentiostat. The EIS analyses are procured at 1 MHz to 100 mHz. The CV curves are measured between 0.01 and 1.2 V at a scan rate of 0.05 mV $s^{-1}$.

## Electrochemical Results

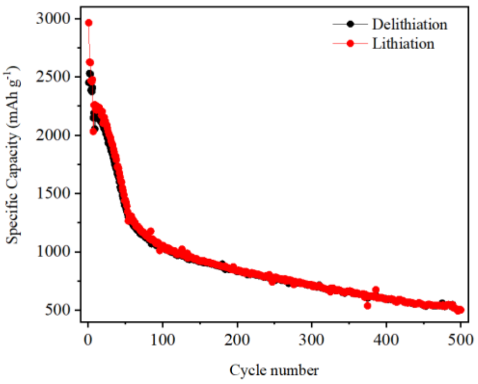


**Figure S1.** GCD at C/5 of Si/CMC-SBR electrode.
